# *rac*-4,5-*trans*-Di­bromo-9,9-di­chloro-*cis*-bi­cyclo[6.1.0]nona­ne

**DOI:** 10.1107/S2414314625005735

**Published:** 2025-06-27

**Authors:** Heiner Detert, Dieter Schollmeyer

**Affiliations:** a University of Mainz, Department of Chemistry, Duesbergweg 10-14, 55099 Mainz, Germany; Goethe-Universität Frankfurt, Germany

**Keywords:** crystal structure, tetra­halogenated bi­cyclo­nona­ne

## Abstract

The crystal structure of the title tetra­halogenated bi­cyclo­nonane, C_9_H_12_Br_2_Cl_2_, is reported. The mol­ecule adopts a distorted twist-chair conformation. The cyclo­propane ring is almost parallel to the plane formed by the four methyl­ene carbon atoms.

## Abstract 2

The crystal structure of a tetra­halogenated bi­cyclo­nonane, C_9_H_12_Br_2_Cl_2_, is reported. The mol­ecule adopts a distorted twist-chair conformation. The cyclo­propane ring is almost parallel to the plane formed by the four methyl­ene carbon atoms.

## Graphical abstract

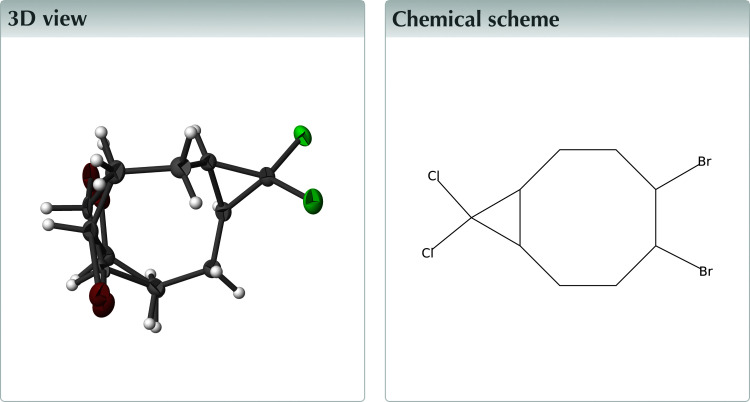

## Structure description

**Figure 1:**
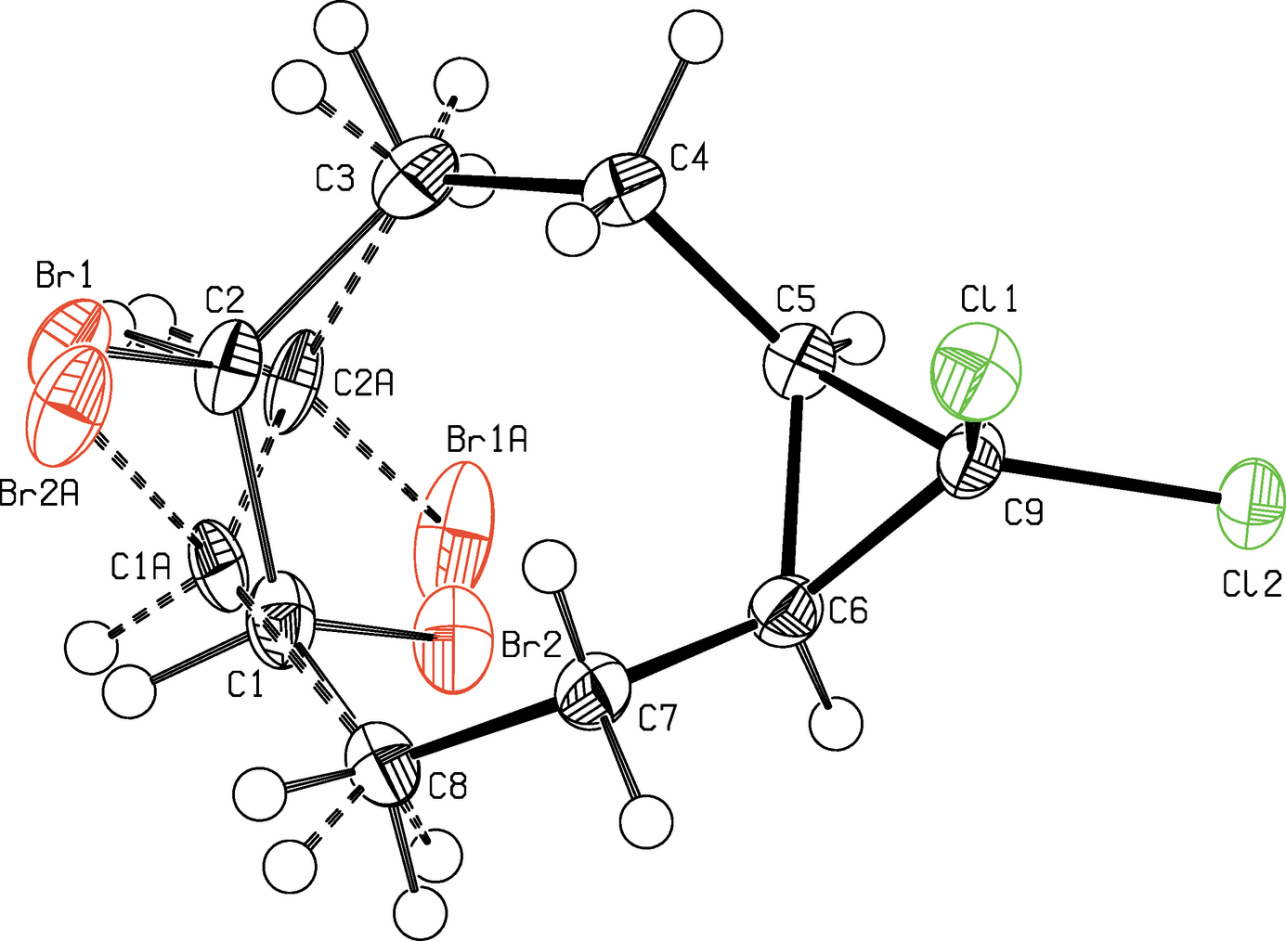
View (Spek, 2009 ▸) of the title compound. Displacement ellipsoids are drawn at the 50% probability level. The minor occupied component is drawn with dashed lines.

**Figure 2:**
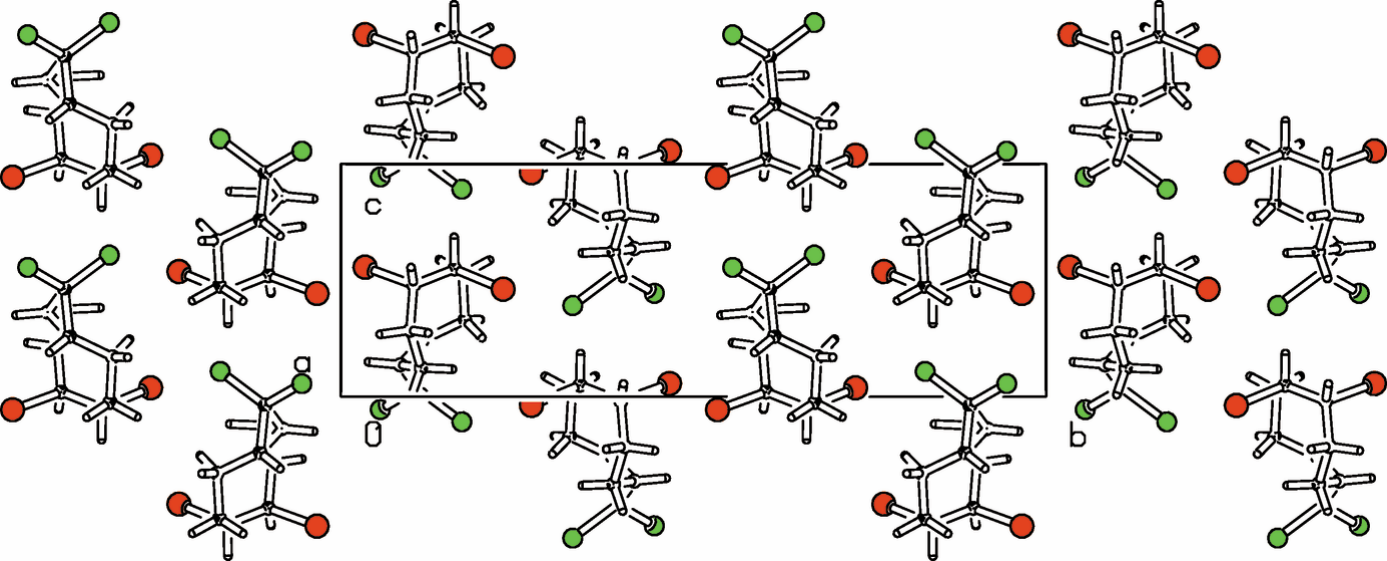
Partial packing diagram (Spek, 2009 ▸), viewed along the *a*-axis direction. The minor occupied component is omitted.

The title compound, C_9_H_12_Br_2_Cl_2_ (Fig. 1 ▸), was prepared as part of a project focusing on medium-sized bicyclic cyclo­alkynes (Meier *et al.*, 1987 ▸; Detert & Meier, 1997 ▸). Both enanti­omers [*(S,S)* and *(R,R-)*] occupy the same positions in the crystal, resulting in disorder on C1 and C2, but the ratio is 0.638 (9)/0.362 (9). The eight-membered ring adopts a distorted twist-chair conformation and the annulated cyclo­propane group is in an *exo*-position. The four methyl­ene carbons (C3, C4, C7, C8) lie in a common plane. This plane is nearly parallel to the cyclo­propane ring, their normals enclose a small angle of only 0.5 (3)°. The bond lengths of the bromine-bound carbon atoms are significantly different: C1—C8: 1.487 (7) Å; C2—C3: 1.567 (7) Å. Furthermore, the C—C—C bond angles are opened to 120.5 (5)° (C2—C8—C1) and even 121.8 (5)° (C1—C2—C3). The same holds for the minor occupied sites. The packing is shown in Fig. 2 ▸.

## Synthesis and crystallization

The title compound, first mentioned by Fray (1963 ▸), was prepared by careful addition of bromine to 9.9-di­chloro­bicyclo­[6.1.0]non-4-ene. Crystals were grown by slow evaporation of a solution in chloro­form and propanol-2 to yield colorless crystals with a m.p. of 336–340 K. The annotation of the NMR signals follows IUPAC nomenclature. ^1^H-NMR (200 MHz, CDCl_3_): 9.1 (*bs*, 1 H, OH), 2.75 (*t*, 2 H, *J* = 6.1 Hz), 2.37 (*t*, 2 H, *J* = 6 Hz), 2.20-1.95 (*m*, 6 H, 3,4,7-H), 1.80 (*m*, 4 H, 8,9-H); ^13^C-NMR (100 MHz, CDCl_3_): 160.3 (C=N), 84.9, 83.4 (C-5, C-6), 26.1, 24.3, 23.9 (C-3,8,9), 19.6, 18.3 (C-4, 7).

## Refinement

Crystal data, data collection and structure refinement details are summarized in Table 1 ▸.

## Supplementary Material

Crystal structure: contains datablock(s) I, global. DOI: 10.1107/S2414314625005735/bt4172sup1.cif

Structure factors: contains datablock(s) I. DOI: 10.1107/S2414314625005735/bt4172Isup2.hkl

Supporting information file. DOI: 10.1107/S2414314625005735/bt4172Isup3.cml

CCDC reference: 2466895

Additional supporting information:  crystallographic information; 3D view; checkCIF report

## Figures and Tables

**Table 1:** Experimental details

Crystal data	
Chemical formula	C_9_H_12_Br_2_Cl_2_
*M*_r_	350.91
Crystal system, space group	Monoclinic, *P*2_1_/*c*
Temperature (K)	120
*a*, *b*, *c* (Å)	7.2027 (3), 22.1157 (10), 7.5534 (3)
β (°)	105.517 (3)
*V* (Å^3^)	1159.35 (9)
*Z*	4
Radiation type	Mo *K*α
μ (mm^−1^)	7.41
Crystal size (mm)	0.50 × 0.46 × 0.13

Data collection	
Diffractometer	Stoe *IPDS* 2T
Absorption correction	Integration [*X-RED32* (Stoe & Cie, 2020 ▸) absorption correction by Gaussian integration]
*T*_min_, *T*_max_	0.069, 0.358
No. of measured, independent and observed [*I* > 2σ(*I*)] reflections	6364, 2766, 2469
*R*_int_	0.034
(sin θ/λ)_max_ (Å^−1^)	0.659

Refinement	
*R*[*F*^2^ > 2σ(*F*^2^)], *wR*(*F*^2^), *S*	0.040, 0.100, 1.19
No. of reflections	2766
No. of parameters	155
H-atom treatment	H-atom parameters constrained
Δρ_max_, Δρ_min_ (e Å^−3^)	0.60, −0.40

Computer programs: *X-AREA WinXpose*, *Recipe* and *Integrate* (Stoe & Cie, 2020 ▸), *SHELXT2014* (Sheldrick, 2015*a* ▸), *SHELXL2019/2* (Sheldrick, 2015*b* ▸) and *PLATON* (Spek, 2009 ▸).

## full crystallographic data

### rac-4,5-trans-Dibromo-9,9-dichloro-cis-bicyclo[6.1.0]nonane Crystal data

**Table:**

C_9_H_12_Br_2_Cl_2_	*F*(000) = 680
*M**_r_* = 350.91	*D*_x_ = 2.010 Mg m^−^^3^
Monoclinic, *P*2_1_/*c*	Mo *K*α radiation, λ = 0.71073 Å
*a* = 7.2027 (3) Å	Cell parameters from 9471 reflections
*b* = 22.1157 (10) Å	θ = 2.8–28.4°
*c* = 7.5534 (3) Å	µ = 7.41 mm^−^^1^
β = 105.517 (3)°	*T* = 120 K
*V* = 1159.35 (9) Å^3^	Plate, colorless
*Z* = 4	0.50 × 0.46 × 0.13 mm

### rac-4,5-trans-Dibromo-9,9-dichloro-cis-bicyclo[6.1.0]nonane Data collection

**Table:**

Stoe IPDS 2T diffractometer	2766 independent reflections
Radiation source: sealed X-ray tube, 12x0.4mm long-fine focus	2469 reflections with *I* > 2σ(*I*)
Detector resolution: 6.67 pixels mm^-1^	*R*_int_ = 0.034
rotation method, ω scans	θ_max_ = 27.9°, θ_min_ = 2.9°
Absorption correction: integration [X-Red32 (Stoe & Cie, 2020) absorption correction by Gaussian integration]	*h* = −9→9
*T*_min_ = 0.069, *T*_max_ = 0.358	*k* = −29→29
6364 measured reflections	*l* = −9→9

### rac-4,5-trans-Dibromo-9,9-dichloro-cis-bicyclo[6.1.0]nonane Refinement

**Table:**

Refinement on *F*^2^	Primary atom site location: dual
Least-squares matrix: full	Hydrogen site location: inferred from neighbouring sites
*R*[*F*^2^ > 2σ(*F*^2^)] = 0.040	H-atom parameters constrained
*wR*(*F*^2^) = 0.100	*w* = 1/[σ^2^(*F*_o_^2^) + (0.0367*P*)^2^ + 2.8083*P*] where *P* = (*F*_o_^2^ + 2*F*_c_^2^)/3
*S* = 1.19	(Δ/σ)_max_ = 0.001
2766 reflections	Δρ_max_ = 0.60 e Å^−^^3^
155 parameters	Δρ_min_ = −0.39 e Å^−^^3^
0 restraints	

### rac-4,5-trans-Dibromo-9,9-dichloro-cis-bicyclo[6.1.0]nonane Special details

**Table:**

Geometry. All esds (except the esd in the dihedral angle between two l.s. planes) are estimated using the full covariance matrix. The cell esds are taken into account individually in the estimation of esds in distances, angles and torsion angles; correlations between esds in cell parameters are only used when they are defined by crystal symmetry. An approximate (isotropic) treatment of cell esds is used for estimating esds involving l.s. planes.
Refinement. Hydrogen atoms attached to carbons were placed at calculated positions and were refined in the riding-model approximation with C_methylene_–H = 0.99 Å or C_tertiary_–H = 1.00 Å, and with *U*_iso_(H) = 1.2 *U*_eq_(C).

### rac-4,5-trans-Dibromo-9,9-dichloro-cis-bicyclo[6.1.0]nonane Fractional atomic coordinates and isotropic or equivalent isotropic displacement parameters (Å^2^)

**Table:**

	*x*	*y*	*z*	*U*_iso_*/*U*_eq_	Occ. (<1)
Cl1	0.67566 (14)	0.67134 (4)	0.60825 (12)	0.0288 (2)	
Cl2	0.83981 (13)	0.55561 (4)	0.55570 (12)	0.02610 (19)	
Br1	0.0626 (5)	0.72992 (15)	0.0378 (5)	0.0261 (4)	0.638 (9)
Br2	0.2483 (2)	0.53409 (6)	−0.0565 (4)	0.0289 (4)	0.638 (9)
C1	0.1135 (9)	0.6031 (4)	0.0199 (9)	0.0243 (14)	0.638 (9)
H1	−0.023418	0.599338	−0.054723	0.029*	0.638 (9)
C2	0.1880 (9)	0.6610 (3)	−0.0491 (8)	0.0232 (13)	0.638 (9)
H2	0.130604	0.660373	−0.185198	0.028*	0.638 (9)
Br1A	0.2998 (9)	0.5459 (2)	−0.1082 (6)	0.0393 (12)	0.362 (9)
Br2A	0.0345 (11)	0.7209 (3)	0.0592 (9)	0.0336 (10)	0.362 (9)
C1A	0.0875 (14)	0.6337 (7)	0.0252 (13)	0.021 (2)	0.362 (9)
H1A	−0.033261	0.617909	−0.061182	0.026*	0.362 (9)
C2A	0.2366 (16)	0.6317 (6)	−0.0823 (13)	0.024 (2)	0.362 (9)
H2A	0.167411	0.645242	−0.209057	0.028*	0.362 (9)
C3	0.4082 (6)	0.67226 (18)	−0.0243 (5)	0.0287 (8)	
H3A	0.457248	0.639468	−0.088844	0.034*	0.638 (9)
H3B	0.422031	0.710584	−0.087348	0.034*	0.638 (9)
H3C	0.360886	0.713840	−0.057962	0.034*	0.362 (9)
H3D	0.492984	0.662025	−0.103791	0.034*	0.362 (9)
C4	0.5379 (5)	0.67580 (16)	0.1709 (5)	0.0242 (7)	
H4A	0.657883	0.697353	0.169044	0.029*	
H4B	0.472136	0.699838	0.246652	0.029*	
C5	0.5899 (5)	0.61482 (15)	0.2597 (5)	0.0192 (6)	
H5	0.653803	0.586721	0.190526	0.023*	
C6	0.4614 (5)	0.58393 (15)	0.3632 (5)	0.0206 (7)	
H6	0.454627	0.538915	0.350552	0.025*	
C7	0.2773 (5)	0.61350 (17)	0.3764 (5)	0.0237 (7)	
H7A	0.295303	0.657907	0.382082	0.028*	
H7B	0.248641	0.600487	0.491711	0.028*	
C8	0.1061 (5)	0.59791 (18)	0.2141 (5)	0.0266 (7)	
H8A	0.070570	0.555553	0.232062	0.032*	0.638 (9)
H8B	−0.002787	0.623390	0.226302	0.032*	0.638 (9)
H8C	−0.013285	0.604977	0.252293	0.032*	0.362 (9)
H8D	0.112053	0.554131	0.188845	0.032*	0.362 (9)
C9	0.6542 (5)	0.60790 (15)	0.4640 (5)	0.0201 (6)	

### rac-4,5-trans-Dibromo-9,9-dichloro-cis-bicyclo[6.1.0]nonane Atomic displacement parameters (Å^2^)

**Table:**

	*U*^11^	*U*^22^	*U*^33^	*U*^12^	*U*^13^	*U*^23^
Cl1	0.0330 (5)	0.0250 (4)	0.0246 (4)	−0.0022 (3)	0.0013 (3)	−0.0062 (3)
Cl2	0.0220 (4)	0.0268 (4)	0.0258 (4)	0.0037 (3)	0.0001 (3)	0.0047 (3)
Br1	0.0348 (8)	0.0208 (6)	0.0219 (8)	0.0074 (5)	0.0063 (5)	0.0023 (5)
Br2	0.0291 (5)	0.0228 (4)	0.0295 (7)	0.0041 (3)	−0.0016 (4)	−0.0071 (4)
C1	0.022 (3)	0.019 (3)	0.027 (3)	0.004 (2)	−0.002 (2)	−0.004 (3)
C2	0.025 (3)	0.025 (3)	0.017 (3)	0.007 (2)	0.001 (2)	0.002 (2)
Br1A	0.0497 (17)	0.0319 (11)	0.0262 (10)	0.0215 (11)	−0.0075 (11)	−0.0111 (9)
Br2A	0.044 (2)	0.032 (2)	0.0244 (13)	0.0204 (15)	0.0094 (10)	0.0019 (12)
C1A	0.013 (4)	0.030 (7)	0.016 (4)	−0.001 (5)	−0.005 (3)	−0.005 (5)
C2A	0.029 (5)	0.027 (6)	0.009 (4)	0.010 (4)	−0.004 (4)	0.002 (4)
C3	0.032 (2)	0.0309 (19)	0.0226 (17)	−0.0001 (15)	0.0064 (15)	0.0088 (15)
C4	0.0259 (18)	0.0212 (16)	0.0256 (17)	−0.0017 (13)	0.0071 (14)	0.0047 (13)
C5	0.0205 (16)	0.0189 (15)	0.0178 (15)	0.0017 (12)	0.0046 (12)	0.0003 (12)
C6	0.0218 (17)	0.0196 (15)	0.0187 (15)	−0.0025 (13)	0.0025 (13)	0.0018 (12)
C7	0.0220 (17)	0.0286 (17)	0.0208 (16)	0.0011 (14)	0.0062 (13)	0.0060 (14)
C8	0.0181 (16)	0.0328 (19)	0.0270 (17)	−0.0009 (14)	0.0027 (14)	0.0034 (15)
C9	0.0210 (16)	0.0191 (15)	0.0183 (15)	0.0019 (12)	0.0017 (13)	0.0029 (12)

### rac-4,5-trans-Dibromo-9,9-dichloro-cis-bicyclo[6.1.0]nonane Geometric parameters (Å, º)

**Table:**

Cl1—C9	1.758 (3)	C3—H3C	0.9900
Cl2—C9	1.763 (3)	C3—H3D	0.9900
Br1—C2	1.972 (9)	C4—C5	1.508 (5)
Br2—C1	1.975 (9)	C4—H4A	0.9900
C1—C8	1.487 (7)	C4—H4B	0.9900
C1—C2	1.533 (10)	C5—C9	1.495 (5)
C1—H1	1.0000	C5—C6	1.524 (5)
C2—C3	1.567 (7)	C5—H5	1.0000
C2—H2	1.0000	C6—C9	1.491 (5)
Br1A—C2A	1.973 (14)	C6—C7	1.505 (5)
Br2A—C1A	1.997 (18)	C6—H6	1.0000
C1A—C2A	1.510 (17)	C7—C8	1.528 (5)
C1A—C8	1.606 (12)	C7—H7A	0.9900
C1A—H1A	1.0000	C7—H7B	0.9900
C2A—C3	1.495 (12)	C8—H8A	0.9900
C2A—H2A	1.0000	C8—H8B	0.9900
C3—C4	1.522 (5)	C8—H8C	0.9900
C3—H3A	0.9900	C8—H8D	0.9900
C3—H3B	0.9900		
			
C8—C1—C2	120.5 (6)	C5—C4—H4B	108.8
C8—C1—Br2	112.3 (4)	C3—C4—H4B	108.8
C2—C1—Br2	107.6 (5)	H4A—C4—H4B	107.7
C8—C1—H1	105.0	C9—C5—C4	121.5 (3)
C2—C1—H1	105.0	C9—C5—C6	59.2 (2)
Br2—C1—H1	105.0	C4—C5—C6	121.1 (3)
C1—C2—C3	121.8 (5)	C9—C5—H5	114.7
C1—C2—Br1	107.4 (5)	C4—C5—H5	114.7
C3—C2—Br1	112.1 (4)	C6—C5—H5	114.7
C1—C2—H2	104.6	C9—C6—C7	121.9 (3)
C3—C2—H2	104.6	C9—C6—C5	59.4 (2)
Br1—C2—H2	104.6	C7—C6—C5	120.4 (3)
C2A—C1A—C8	124.1 (10)	C9—C6—H6	114.7
C2A—C1A—Br2A	106.6 (10)	C7—C6—H6	114.7
C8—C1A—Br2A	109.6 (6)	C5—C6—H6	114.7
C2A—C1A—H1A	105.0	C6—C7—C8	112.6 (3)
C8—C1A—H1A	105.0	C6—C7—H7A	109.1
Br2A—C1A—H1A	105.0	C8—C7—H7A	109.1
C3—C2A—C1A	118.5 (10)	C6—C7—H7B	109.1
C3—C2A—Br1A	114.3 (7)	C8—C7—H7B	109.1
C1A—C2A—Br1A	107.3 (10)	H7A—C7—H7B	107.8
C3—C2A—H2A	105.2	C1—C8—C7	122.7 (4)
C1A—C2A—H2A	105.2	C7—C8—C1A	117.4 (5)
Br1A—C2A—H2A	105.2	C1—C8—H8A	106.7
C2A—C3—C4	124.0 (4)	C7—C8—H8A	106.7
C4—C3—C2	117.6 (3)	C1—C8—H8B	106.7
C4—C3—H3A	107.9	C7—C8—H8B	106.7
C2—C3—H3A	107.9	H8A—C8—H8B	106.6
C4—C3—H3B	107.9	C7—C8—H8C	108.0
C2—C3—H3B	107.9	C1A—C8—H8C	108.0
H3A—C3—H3B	107.2	C7—C8—H8D	108.0
C2A—C3—H3C	106.3	C1A—C8—H8D	108.0
C4—C3—H3C	106.3	H8C—C8—H8D	107.2
C2A—C3—H3D	106.3	C6—C9—C5	61.4 (2)
C4—C3—H3D	106.3	C6—C9—Cl1	121.0 (3)
H3C—C3—H3D	106.4	C5—C9—Cl1	120.6 (2)
C5—C4—C3	113.6 (3)	C6—C9—Cl2	118.2 (2)
C5—C4—H4A	108.8	C5—C9—Cl2	117.7 (2)
C3—C4—H4A	108.8	Cl1—C9—Cl2	110.36 (19)
			
C8—C1—C2—C3	84.4 (8)	C9—C6—C7—C8	158.3 (3)
Br2—C1—C2—C3	−46.0 (7)	C5—C6—C7—C8	87.5 (4)
C8—C1—C2—Br1	−46.9 (7)	C2—C1—C8—C7	−49.5 (8)
Br2—C1—C2—Br1	−177.3 (3)	Br2—C1—C8—C7	78.9 (5)
C8—C1A—C2A—C3	−82.3 (15)	C6—C7—C8—C1	−49.5 (6)
Br2A—C1A—C2A—C3	46.3 (10)	C6—C7—C8—C1A	−79.0 (7)
C8—C1A—C2A—Br1A	48.9 (11)	C2A—C1A—C8—C7	60.9 (13)
Br2A—C1A—C2A—Br1A	177.6 (5)	Br2A—C1A—C8—C7	−66.4 (7)
C1A—C2A—C3—C4	52.0 (13)	C7—C6—C9—C5	−108.9 (3)
Br1A—C2A—C3—C4	−76.0 (7)	C7—C6—C9—Cl1	1.5 (4)
C1—C2—C3—C4	−62.2 (7)	C5—C6—C9—Cl1	110.4 (3)
Br1—C2—C3—C4	67.1 (5)	C7—C6—C9—Cl2	143.1 (3)
C2A—C3—C4—C5	44.4 (8)	C5—C6—C9—Cl2	−108.0 (3)
C2—C3—C4—C5	79.2 (5)	C4—C5—C9—C6	109.9 (4)
C3—C4—C5—C9	−158.2 (3)	C4—C5—C9—Cl1	−1.2 (5)
C3—C4—C5—C6	−87.6 (4)	C6—C5—C9—Cl1	−111.1 (3)
C4—C5—C6—C9	−110.5 (4)	C4—C5—C9—Cl2	−141.4 (3)
C9—C5—C6—C7	111.4 (4)	C6—C5—C9—Cl2	108.7 (3)
C4—C5—C6—C7	0.9 (5)		
